# Improving Contagion and Horizontal Transmission of Entomopathogenic Fungi by the White-Spotted Longicorn Beetle, *Anoplophora malasiaca*, with Help of Contact Sex Pheromone

**DOI:** 10.3390/insects12050383

**Published:** 2021-04-26

**Authors:** Nao Fujiwara-Tsujii, Hiroe Yasui

**Affiliations:** Chemical Ecology Group, Central Region Agricultural Research Center, NARO, Tsukuba, Ibaraki 305-8666, Japan; naoki99@affrc.go.jp

**Keywords:** arrestant, Biolisa Kamikiri SLIM, microbial insecticide, mating, infection, control

## Abstract

**Simple Summary:**

The white-spotted longicorn beetle, *Anoplophora malasiaca*, is one of the most destructive pests of many fruits and street trees. Effective controls are needed because the effect of marketed insecticides is limited. Entomopathogenic fungi offer a solution, combination with the beetles’ contact sex pheromone. The surface of the female body is covered with contact sex pheromone, which we extracted. Males held onto a glass model coated with female extract for 5 h, but males held onto one without extract for <0.3 h. Males that held onto coated model, attached to fabric impregnated with an entomopathogenic fungus, *Beauveria brongniartii*, picked up significant fungi. The fungi were then transferred to females during mating. Our results indicate that a combination of contact pheromone with a pathogen could improve entomopathogenic infection of both male and female beetles.

**Abstract:**

The white-spotted longicorn beetle, *Anoplophora malasiaca*, is one of the most destructive pests of horticultural crops and street trees. Effective controls are needed because the effect of marketed insecticides is limited. Entomopathogenic fungi offer a solution, and improving the rate of infection would be a breakthrough in this beetle’s control. The combination of pathogenic fungi and the beetle’s contact sex pheromone was suggested. The surface of the female body is covered with contact sex pheromone, which elicit male mating behavior. To develop a method for the practical control of this beetle, we evaluated the arrestant activity of female extract containing contact pheromone coated on a black glass model. Males presented with a coated model held on for 5 h (mean) during an 8-h experiment. In contrast, males presented with a control model held on for <0.3 h. Males that held onto coated models attached to fabric impregnated with conidia of the fungus *Beauveria brongniartii* picked up much conidia, which they then passed on to females during mating.

## 1. Introduction

The white-spotted longicorn beetle, *Anoplophora malasiaca* (Thomson) (Coleoptera: Cerambycidae), has a very wide range of host plants exceeding 100 species [1]. It is one of the most destructive pests of horticultural crops and street trees including citrus, blueberry, plane tree, maple, and willow [2,3]. This species is distributed in Japan [3] and effective controls are needed because the effect of marketed chemical insecticides is limited. Microbial insecticides offer a solution [4,5,6,7,8,9,10]. Compared to chemical insecticides, entomopathogenic ones pose minimal risk to nontarget organisms and are environmentally friendly [11]. Studies performed with fabric bands impregnated with conidia of entomopathogenic fungi are reported [8,9,10]. In Japan, non-woven fabric band impregnated with conidia of an entomopathogenic fungus, *Beauveria brongniartii,* named “Biolisa Kamikiri SLIM”, is commercially available [12]. A fabric band is looped around the base of the tree trunk or a main branch, and adults become infected through direct contact with conidia on the fabric, which relies on chance.

Fungal infections can be transferred during mating: an infected beetle of a closely related species, *A. glabripennis,* can transfer an infection to its mating partner, particularly if the infected beetle is male [7,13]. In other Coleoptera, horizontal transmission has been reported in *Psacothea hilaris* [14] and *Cylas formicarius* [15]. Improving the infection rate would be a breakthrough in beetle control, especially as microbial insecticides are environmentally friendly and non-toxic to humans. Other studies that address the combination of attractant pheromones and entomopathogens in Coleoptera revealed effective transmission of the pathogen [15,16].

The entire composition of the contact sex pheromone of *A. malasiaca* female was recently elucidated [17]. The surface of the female body is covered with contact sex pheromone, which is composed of three chemical groups: aliphatic hydrocarbons, ketones, and lactones [18,19,20,21,22]. When at least one component of each group was mixed together and presented to males, the males responded to the mixture in the same way as to crude female extract [18,19,20]. In our recent work, all synthetic pheromone blends elicited pheromonal activity to the same degree as did crude female extract [17]. During bioassays investigating pheromone candidates, we observed that males held strongly onto glass models of females and would not let go. This strong attachment gave us the idea to test a combination of entomopathogenic fungi and contact sex pheromone. If the contact pheromone has arrestant activity, it will increase the duration of contact and thus the rate of infection. Since the synthesis of the lactone components of the contact pheromone is complex, we used crude female extract to evaluate the activity. To develop a method for the practical control of this beetle, we tested whether the combination of “Biolisa Kamikiri SLIM” and contact pheromone improves the dose and spread of infection beyond the use of fungi alone.

## 2. Materials and Methods 

### 2.1. Insect Collection and Laboratory Rearing to Adults 

*Anoplophora malasiaca* adults were collected by hand from mandarin orange (*Citrus unshiu* Marc.) groves on Kunisaki Peninsula, Oita Prefecture, Japan, in mid-June 2016. Beetles were individually reared in clear plastic cups (~11 cm diameter × 9.5 cm height) at 25 °C under a 15 L:9 D photoperiod, illuminated by fluorescent lamps. Each beetle was fed *C. unshiu* branches collected from the sampling sites. All cut branches were stored at 5 °C and used within 10 days. Eggs were obtained from 200 females collected in the field. Eggs laid on branches in the laboratory were collected and the hatched larvae were individually reared to adults on an artificial diet (Silkmate 2S, mulberry leaf–based diet; Nihon Nosan Kogyo, Yokohama, Japan). Emerged adults were individually held in clear plastic cups (as above). Adults started feeding on willow branches 1 week after emergence and were reared on willow for 3 weeks. 

### 2.2. Extraction of Female Contact Pheromone

After 3 weeks of feeding, females were frozen and stored at −30 °C. Because extracts of willow fed females were more attractive to *A. malasiaca* males than that of citrus–fed females, and mature female extract is more attractive than immature female extract [23], we used extract of 3–week–willow–fed–female for bioassay. The elytra of 10 females were removed and placed in 15 mL diethyl ether (1.5 mL/female). After 5 min at room temperature, the extract was decanted. Elytra were rinsed twice with 15 mL ether, and the rinses were added to the extract. Ether was removed from the extracts under reduced pressure at <30 °C, and the resulting residue (the “female extract”) was dissolved in 200 μL *n*-hexane and stored at −30 °C until use. Solvents were distilled just before use.

### 2.3. Recording Male Behavior to Evaluate Arrestant Activity of Female Extract 

**Figure 1:**
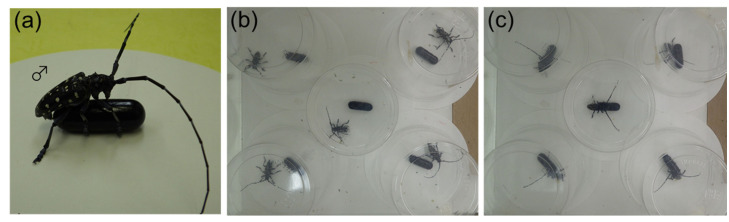
*Anoplophora malasiaca* males. (**a**) Male holding a glass model coated with female extract and bending his abdomen toward it. (**b**) Males with solvent-only glass models. All males ignored the models. (**c**) Males with models coated with female extract. Most males kept hold of the models.

Behavioral assays were conducted between 09:00 and 17:00 at 25 °C (8 h recording, 4 h after lights on). A capsule-shaped black glass model (12 mm diameter × 35 mm length) was used as a female dummy [24]. It was affixed with a small piece of adhesive tape to the center of a filter paper disc (15 cm diameter, Toyo No. 2, Toyo Roshi Kaisha, Tokyo, Japan) and coated with female extract (1 female equivalent, fe), dissolved in ca. 20 μL *n*-hexane. A male beetle was placed with a model coated with female extract or just solvent and covered with a clear plastic cup (as above). We tested 5 males separately at the same time (Figure 1b,c). Male behavior was recorded every 10 min with a time-lapse camera. When a male was seen to be holding the model, the holding period was calculated as (no. of recorded of “holding” × 10) min. The longest holding period was recorded. The sample sizes were 20 for female extract and 5 for solvent only.

### 2.4. Conidial Adherence to Males

**Figure 2:**
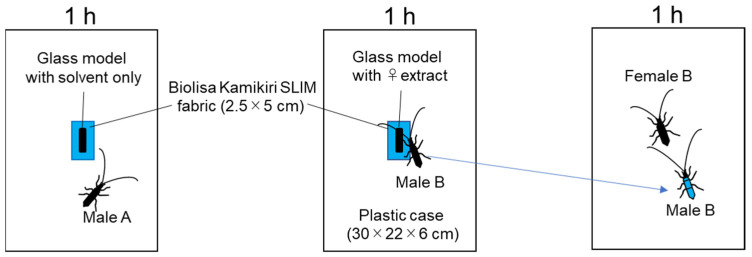
A male (A, B) was placed with a glass model on “Biolisa Kamikiri SLIM” fabric in a plastic box for 1 h. Male B was placed with female B in another case for another 1 h.

“Biolisa Kamikiri SLIM” (Idemitsu Agri Co., Ltd. Tokyo, Japan) non-woven fabric (4 mm thick) was cut into pieces of 2.5 cm × 5.0 cm. A glass model coated with 1 fe extract or solvent only was attached to its center and placed at the center of a plastic box (30 cm × 22 cm × 6 cm; Figure 1). A male was introduced near the model and left for 1 h. Males were introduced near a glass model treated with solvent only (Male A, *n* = 9, Figure 2 left) or treated with female extract (Male B, *n* = 10, Figure 2 middle). The fabric size was larger than the model, so the male antennae and legs were encountered the fabric first. All males placed with coated glass models held the models and bend their abdomens toward them. After 1 h, each male was ultrasonicated in 15 mL of 0.02% polyoxyethylene (20) sorbitan monopalmitate (Wako Pure Chemical Industry, Osaka, Japan, Tween 40, surfactant) in water for 2 min, and the mean number of conidia in the solution (number of conidia/mm^3^) were counted by hemocytometer under a microscope, then calculated the number of conidia released from the each male.

### 2.5. Conidial Adherence to Females That Copulated with Infected Males

A male prepared as in Section 2.4 (Male B) was placed together with a female (Female B, *n* = 10) in a plastic box (30 cm × 22 cm × 6 cm) for 1 h (Figure 2 right). All Male B were used only once. All males used in this assay held the female and bent their abdomen toward the female. After 1 h, Female B was ultrasonicated as in Section 2.4, and conidia were counted.

### 2.6. Statistical Analysis

The calculated longest holding time (*x* min) was analyzed by Student’s *t*-test at *p* < 0.01. Individual conidial number (*y*) was tested by one-way analysis of variance (ANOVA), and analyzed by Tukey’s multiple comparison at *p* < 0.05. 

## 3. Results

### 3.1. Arrestant Effect of Female Extract on Males

When a glass model coated with female extract was presented to males, almost all males persistently tried to copulate with it (Figure 1a). During 8 h recording, males presented with an solvent only model walked around the floor and on the wall of the plastic cup (Figure 1b). However, most males presented with coated model held onto the model for a long time, and if they left it, they returned to it and held it again (Figure 1c).

**Figure 3:**
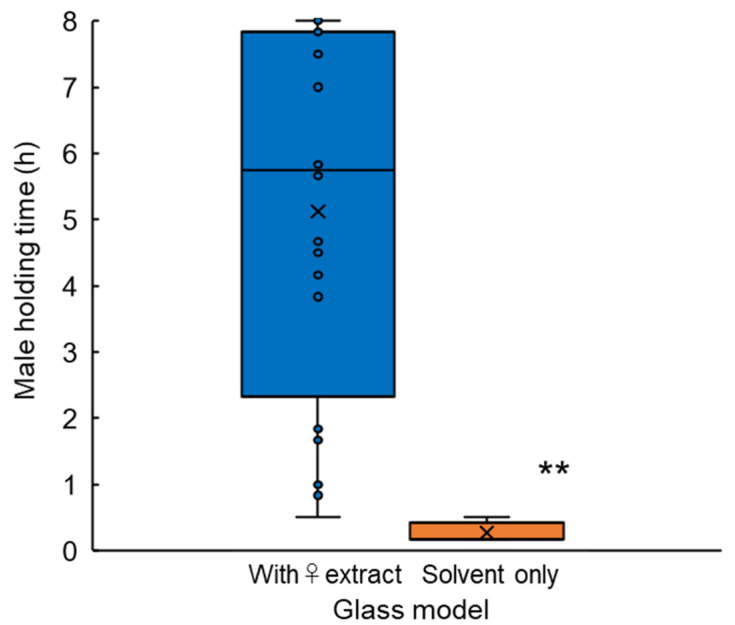
Male longest arrested time on a glass model coated with or without female extract during 8-h experiment. Box-plots: middle line, mean; boxes, quartiles; whiskers, maximum and minimum values. ○ Data points; × average. ** Significantly different at *p* < 0.01 (Student’s *t*-test).

Extract-coated glass models arrested males for an average of 5.1 h (307.5 ± 36.5 min; mean ± SE, *n* = 20) per 8-h experiment (Figure 3). Maximum arrested time was 8 h that equals to experiment period. On contrast, models without female extract only arrested males less than 0.3 h (16.0 ± 4.0 min, *n* = 5), arrested time was significantly different to that of female extract coated model (Student *t*-test, *p* < 0.01). 

### 3.2. Conidial Adherence to Males with Help of Female Extract

**Figure 4:**
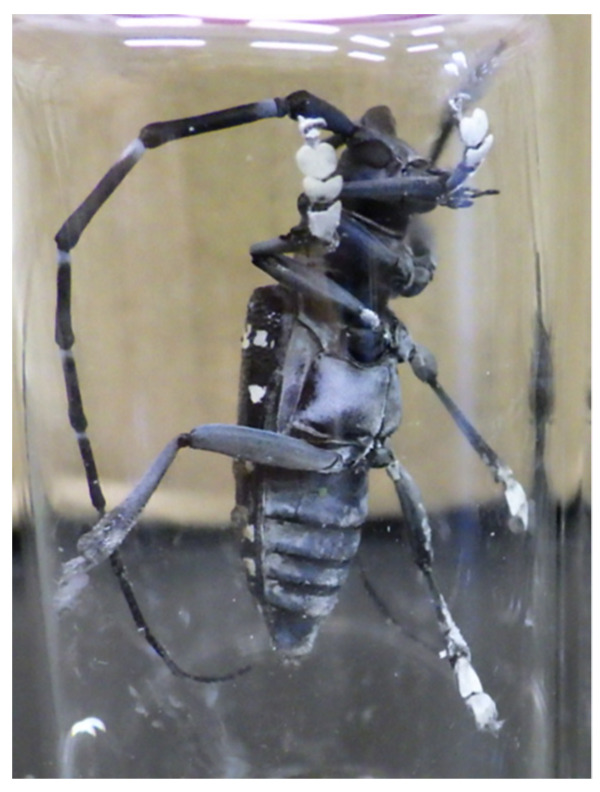
*Anoplophora malasiaca* male B after contact with “Biolisa Kamikiri SLIM” fabric. Foot pads and underside of abdomen are covered with white conidia.

**Figure 5:**
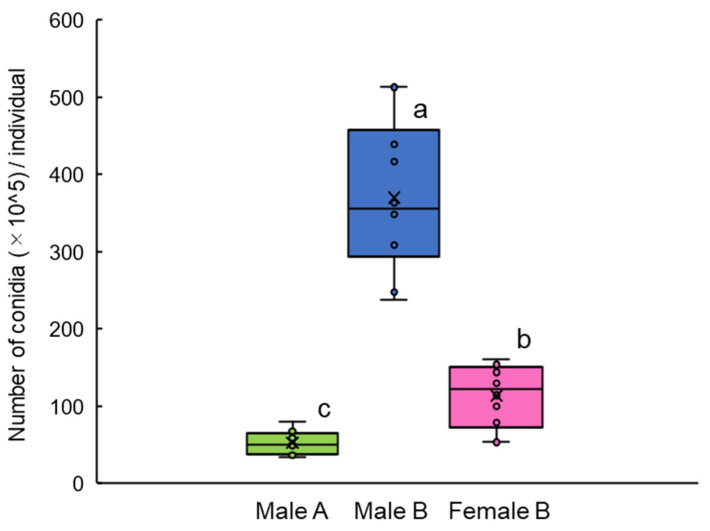
Numbers of conidia attached to adults’ bodies. Male A: placed with a glass model without female extract on “Biolisa Kamikiri SLIM” fabric for 1 h. Male B: placed with a glass model with female extract on “Biolisa Kamikiri SLIM” for 1 h. Female B: placed with Male B for another 1 h. Conidia were washed off in surfactant and counted. Box plots: middle line, mean; boxes, quartiles; maximum and minimum values, whiskers. ○ Data points; × average. Different letters indicate significant differences (Tukey’s test, *p* < 0.05).

To evaluate the effect of the arrestant activity of female extract on infection by the fungus, we compared numbers of conidia on the body surface between males on models with and males on models without female extract on “Biolisa Kamikiri SLIM” fabric (Figure 2, left and center). Males presented with extract-coated models on “Biolisa Kamikiri SLIM” held the models without hesitation. These males acquired significantly more conidia (370.2 × 10^5^ ± 104.4 × 10^5^, mean ± SE; Figure 4 and Figure 5 middle) than those presented with uncoated models on “Biolisa Kamikiri SLIM” (52.7 × 10^5^ ± 15.9 × 10^5^; Tukey’s test, *p* < 0.05; Figure 5 left). The mortality rates after 7 d were 50% with 3 s contact and 90% with 60 s contact (*n* = 10; Figure S1a, χ^2^-test, *p* < 0.06), and was 80% and 100% after 9 d.

### 3.3. Conidial Adherence to Live Females by Mating with Conidia Attached Males

Males acquired conidia in the presence of female extract transferred significantly more conidia to females during mating (Female B, 113.7 × 10^5^ ± 41.3 × 10^5^, Figure 5 right), than acquired by males in the absence of extract (Male A, 52.7 × 10^5^ ± 15.9 × 10^5^; Tukey’s test, *p* < 0.05, Figure 5 left). The mortality of mated adults (both males exposed to “Biolisa Kamikiri SLIM” for 60 s and females that mated with them) after 7 d was 100% (Figure S1b, *n* = 5; 5 of 8 pairs mated). No unexposed males or females of 3 weeks’ rearing died during the 9-day experiment.

## 4. Discussion

Contact sex pheromone of female *A. malasiaca* proved to have arrestant activity against conspecific males. The combination of entomopathogenic fungi and female contact sex pheromone improved both dose and spread of infection compared with fungi alone. A glass model coated with or without female extract was placed on fabric impregnated with conidia of entomopathogenic fungi and presented to males. Crude female extract on a black glass model greatly increased the male’s exposure to the inoculated fabric, and, thus, the number of conidia picked up and later transferred to females during mating. 

Our group previously showed that a capsule-shaped black glass model used as a female dummy and coated with female extract elicits mating behavior by males [24]. Models of other colors or of transparent glass are less attractive. Models without female extract do not elicit male mating behavior.

Bands of non-woven fabric or polyurethane impregnated with *B. brongniartii* conidia are looped around tree stems or branches [9,25], where they infect adult beetles that walk over them, so they must be placed in positions where large numbers of adults pass through [26]. *Anoplophora malasiaca* generally oviposit at and emerged from the base of the host tree trunk, so if the bands are placed below the first branch, not only expecting laying egg female, all newly emerging adults should to be infected as they walk up the tree to feed [9]. The application of a conidial suspension to only abdomen, legs, or antennae of *A. malasiaca* led to high mortality [27]. The mortality of beetles that spent only 3 to 6 s walking on polyurethane foam impregnated with conidia of *B. brongniartii* for 3–6 s was 100% after 15 days [9]. Here, the mortality of beetles that walked on “Biolisa Kamikiri SLIM” for only 3 s was 50% and for 60 s was 90% after 7 days (Figure S1a), and was 80% and 100% after 9 days. When adult beetles of *Capnodis tenebrionis* were stimulated to climb non-woven commercial fiber bands impregnated with *Metarhizium anisopliae* conidia, no significant correlation was detected between the time needed to cross the band and time of death; total mortality rates were 85.7% to 100.0% [10]. In *A. malasiaca*, in contrast, the frequency of touching bands impregnated with *B. brongniartii* conidia might be improved for greater mortality in the field [27]. Here, mortality increased with longer exposure to the fabric, supporting the use of the fabric band in combination with female contact pheromone to enhance infection of this species. No unexposed males or females died during the 9-day experiment. These results and previous reports show that it would be worthwhile for practical control of *A. malasiaca* to improve the frequency of contact with contaminated fabric.

Our results show that the contact pheromone kept the males in contact with the “Biolisa Kamikiri SLIM” fabric for longer, increasing the number of fungal conidia on them (Figure 5). Rather than continuously holding the coated glass models, the males sometimes released the models, walked around them, and held them again. This possible female-guarding behavior thus increases the chance of picking up conidia. Later contact of these infected males with females thus infects the females. Sexual transmission of *B. brongniartii* by mating of the yellow spotted longicorn beetle *P. hilaris* has been reported [14]. So even if females do not come into contact with the “Biolisa Kamikiri SLIM”, they have a chance of infection through mating. At 7 d after mating with males that spent 60 s in contact with conidia, female mortality was 100% (Figure S1b). This means that conidia on male bodies were successfully transferred to females during mating, and that dose enough to kill her. Thus, contact pheromone might improve the infection rate in the field. One practical use of Biolisa Kamikiri SLIM with a contact pheromone is a fabric band on which 1 or 2 glass models coated with female extract (or, in future, synthetic pheromone blend), looped around the base of the trunk, as is used already.

The effectiveness of entomopathogens as biopesticides can be enhanced by their use in combination with chemical control methods. When used together, *M. brunneum* and the insecticide imidacloprid synergistically reduced survival time of *A. glabripennis* adults relative to either alone [28]. However, the rate of sporulation of the fungus was reduced.

Many cerambycid beetles are known to have a contact pheromone [29,30,31,32,33,34,35,36,37]. If these pheromones have arrestant activity, they could be used to increase the duration of contact with entomopathogenic fungi. This strategy might be applied to other important pest beetles.

A volatile pheromone-based attract-and-kill strategy might be made possible by using bait stations containing an attractant along with either a toxicant or an entomopathogen [7,15,16,25]. Sex pheromones have been used as beetle attractants; a sex pheromone trap made with a bottle with exit holes containing conidia of *B. bassiana* was designed and tested to control the sweet potato weevil, *Cylas formicarius* [15]. The use synthetic female sex pheromone was effective for the transmission of *M. brunneum* by male *Agriotes obscurus* click beetles [16]. An attract-and-kill strategy against *A. malasiaca* is not yet realistic because no effective volatile pheromone nor attractant has yet been formulated. Several reports describe the use of chemicals derived from host plants or male volatiles to attract *A. malasiaca* over short distances [38,39,40]. A blend of sesquiterpenes derived from a host plant *C. unshiu* attracted adults in the field, although they landed not on but near lures which impregnated with the blend [38]. This would increase the opportunity for infection if the microbial fabric were set near the lures.

However, once an effective attractant is developed, beetles will be attracted to the fungal bands and become infected. In combination with a contact pheromone, attract-delay-kill strategy could achieve control of *A. malasiaca.*

## 5. Conclusions

Female contact pheromone of *A. malasiaca* arrested conspecific males. In combination with a black glass model coated with female extract and fabric impregnated with conidia of an entomopathogenic fungus, it increased the number of conidia on male bodies and thus mortality. Infected males transferred conidia to the females during mating. This combination could be used to develop a method for the effective control of this beetle.

## Supplementary Materials

The following are available online at https://www.mdpi.com/article/10.3390/insects12050383/s1, Figure S1. Mortality of *A. malasiaca* adults 7 d after contact with “Biolisa Kamikiri SLIM”. (**a**) Adult mortality following 3 or 60 s contact (each *n* = 10, virgin adults, χ^2^-test, *p* < 0.06). (**b**) Mortality of mated adults. “60 sec/mated ♂”: males exposed to “Biolisa Kamikiri SLIM” for 60 s and then mated with females. “mated ♀”: females that mated with males exposed to “Biolisa Kamikiri SLIM” for 60 s just before mating (*n* = 5, 5 of 8 pairs mated)**.**
