# Supplementary material for: Improving Contagion and Horizontal Transmission of Entomopathogenic Fungi by the White-Spotted Longicorn Beetle, Anoplophora malasiaca, with Help of Contact Sex Pheromone
Source: Insects. 2021 Apr 26;12(5):383. doi: 10.3390/insects12050383 (PMC8145553; doi:10.3390/insects12050383)
Supplement: Supplementary file 1 [file insects-12-00383-s001.zip › insects-1153177-supplementary.pdf]

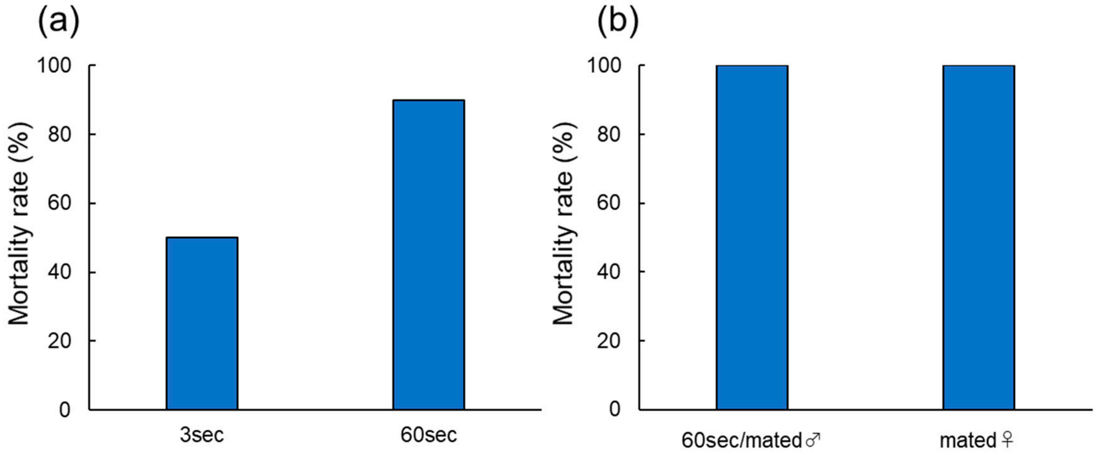

Figure S1. Mortality of *A. malasiaca* adults 7 d after contact with "Biolisa Kamikiri SLIM". (**a**) Adult mortality following 3 or 60 s contact (each $n = 10$, virgin adults, χ^2-test, $p < 0.06$). (**b**) Mortality of mated adults. "60 sec/mated♂": males exposed to "Biolisa Kamikiri SLIM" for 60 s and then mated with females. "mated♀": females that mated with males exposed to "Biolisa Kamikiri SLIM" for 60 s just before mating ($n = 5$, 5 of 8 pairs mated).
